# ‘TXT2BFiT’ a mobile phone-based healthy lifestyle program for preventing unhealthy weight gain in young adults: study protocol for a randomized controlled trial

**DOI:** 10.1186/1745-6215-14-75

**Published:** 2013-03-18

**Authors:** Lana Hebden, Kate Balestracci, Kevin McGeechan, Elizabeth Denney-Wilson, Mark Harris, Adrian Bauman, Margaret Allman-Farinelli

**Affiliations:** 1 School of Molecular Bioscience, University of Sydney, Sydney, NSW 2006, Australia; 2 Sydney School of Public Health, University of Sydney, Sydney, NSW, 2006, Australia; 3 Faculty of Health, University of Technology Sydney, Sydney, NSW, 2008, Australia; 4 Centre for Primary Health Care and Equity, University of New South Wales, Sydney, 2052, Australia

**Keywords:** Young adult, Mobile phone, Text messaging, Intervention studies, Weight loss, Overweight, Primary healthcare

## Abstract

**Background:**

Despite international efforts to arrest increasing rates of overweight and obesity, many population strategies have neglected young adults as a target group. Young adults are at high risk for unhealthy weight gain which tends to persist throughout adulthood with associated chronic disease health risks.

**Methods/design:**

TXT2BFiT is a nine month two-arm parallel-group randomized controlled trial aimed at improving weight management and weight-related dietary and physical activity behaviors among young adults. Participants are recruited via general practice (primary medical care) clinics in Sydney, New South Wales, Australia. All participants receive a mailed resource outlining national physical activity and dietary guidelines and access to the study website. Additional resources accessible to the intervention arm via the study website include Smartphone mobile applications, printable handouts, an interactive healthy weight tracker chart, and a community blog. The study consists of two phases: (1) Intensive phase (weeks 1 to 12): the control arm receives four short message service (SMS) text messages; the intervention arm receives eight SMS messages/week tailored to their baseline stage-of-change, one Email/week, and personalized coaching calls during weeks 0, 2, 5, 8, and 11; and (2) Maintenance phase (weeks 14 to 36): the intervention arm receives one SMS message/month, one Email/month and booster coaching calls during months 5 and 8. A sample of N = 354 (177 per arm) is required to detect differences in primary outcomes: body weight (kg) and body mass index (kg/m^2^), and secondary outcomes: physical activity, sitting time, intake of specific foods, beverages and nutrients, stage-of-change, self-efficacy and participant well-being, at three and nine months. Program reach, costs, implementation and participant engagement will also be assessed.

**Discussion:**

This mobile phone based program addresses an important gap in obesity prevention efforts to date. The method of intervention delivery is via platforms that are highly accessible and appropriate for this population group. If effective, further translational research will be required to assess how this program might operate in the broader community.

**Trial registration:**

Australian New Zealand Clinical Trials Registry ACTRN12612000924853

## Background

Globally, population body mass index (BMI) has been shown to have progressively increased since the 1980s, placing escalating burdens on public health and requiring urgent intervention and policies to reverse this trend [1]. Despite concerted international efforts to arrest increasing rates of overweight and obesity, most strategies have targeted middle-aged adults, children and adolescents [2], while young adults have been largely neglected. A generational effect has arisen, whereby cohorts born between 1960 and 1975 are at greater risk for becoming overweight or obese than previous generations [3-5]. Risk of overweight and obesity has also increased with age up until the mid-50s to mid-60s, with variations between the genders [3,5]. The most rapid weight gain in the life-course has been observed during the early to mid-twenties [6], a life-stage more commonly termed ‘young adulthood’ or ‘emerging adulthood’ [7]. Weight gain during this life-stage has been associated with an increased risk of developing chronic disease risk factors [8-10], and with endometrial cancer [11].

A number of important lifestyle risk factors have been associated with weight gain during this life-stage. Namely, a decline in physical activity [12], an increase in sedentary behavior [13], frequent consumption of fast-food (energy-dense take-away meals) and sugar-sweetened beverages (SSB) [14,15], as well as higher total energy and fat intakes [16]. Another potential risk factor is the inadequate fruit and vegetable intake evident among young adults [17], given the ‘hypo-energetic’ nature of fruit and vegetables and evidence indicating a reduced risk of weight gain with increasing intake [18].

Despite the clear risk for weight gain during young adulthood, evidence to inform effective interventions for this population is lacking [19]. Easy access to treatment has been identified as important in this age group [20], and hence mobile phone-based interventions hold promise for delivering interventions with potentially low resource intensity and wide population reach. At the end of 2011, for every 100 persons there were 86 mobile phone subscriptions globally (122 in developed countries) and 16 mobile broadband subscriptions (51 in developed countries) [21]. In 2012, 74% of 25 to 34 year-olds owned a ‘Smartphone’, compared with 56% of those aged over 13 years [22]. Furthermore, 18 to 29 year-olds send/receive an average of 88 SMS text messages each day - more than triple the volume sent by 30 to 49 year-olds [23].

A pilot randomized controlled trial (RCT) was conducted in the latter half of 2011, with young adults recruited through the tertiary education setting (unpublished). Process findings from this research revealed that the young adults studied preferred set targets for their body weight, with regular weight monitoring, personalized regular contact for accountability, support with planning meals and work/rest/activity schedules, faster operating time of and motivation/reminders to use Smartphone applications, and to receive text messages outside of morning hours. Based on these findings, the program and materials were refined to develop the TXT2BFiT program. This paper reports the protocol for a RCT aimed at (1) testing the efficacy of the TXT2BFiT program by comparing changes in body weight and selected dietary, physical activity and sedentary behaviors among young adults aged 18 to 35 years with changes in a control group, and (2) evaluating program reach, costs, implementation and participant engagement to inform the potential future translation of the program into the broader community.

## Methods/design

Materials and methods of the TXT2BFiT nine month (36 week) parallel-group RCT were approved by the University of Sydney Human Research Ethics Committee in September 2012 (Approval number 15226). The trial is registered with the Australian New Zealand Clinical Trials Registry (ACTRN 12612000924853). It is hypothesized that the tailored TXT2BFiT program will be more effective in improving young adults’ diets, physical activity and weight management, over nine months, compared with controls.

### Participants

Participants are patients of general practice (primary medical care) clinics in the Sydney area, New South Wales, Australia. Eligibility criteria for patients to participate in the study are presented in Table 1. Adults aged 18 to 35 years are selected to include both emerging adulthood (18 to 25 years) and the years until middle-age [3].

**Table 1:** Participant eligibility criteria for participation in the TXT2BFiT trial

**Inclusion criteria**	
1	Body mass index 25.0 to 31.9 kg/m^2 ^or 23.0 to 24.9 kg/m^2^ with reported weight gain > 2 kg over past 12 months.
2	Aged 18 to 35 years.
3	Fruit intake < two serves/day and/or vegetable intake < five serves/day and/or sugar sweetened beverage intake ≥ 1 L/week and/or energy-dense take-away meals > once/week and/or moderate intensity physical activity < 60 minutes/day.
4	Has mobile phone capable of receiving text messages and access to the Internet at least once a week.
**Exclusion criteria**	
1	Pregnant or planning to fall pregnant within the next nine months.
2	Enrolled in an alternate weight loss program.
3	Has lost > 10 kg voluntarily in the past three months.
4	Taking medications that have caused > 2 kg weight gain.
5	Medical condition that precludes following dietary or physical recommendations.
6	History of disordered eating.
7	Does not speak English.

### Procedure

Australian primary health care services were re-organized in July 2011 into independent entities called ‘Medicare Locals’, which are responsible for coordinating primary health care over a specified geographic area. From the Medicare Local, fax and Email invitations are sent to general practitioners (GPs) within the area to partner in the recruitment of patients into the study. GPs returning their expression of interest are then contacted to arrange a time and date for the researchers to attend their practice clinic and to conduct an audit of their electronic patient database in order to identify suitable participants, that is, patients aged 18 to 35 years who have been seen by the GP in the prior 12 months. These patients are mailed a letter from the GP inviting them to participate in the research, along with a consent form and further information about the study. A maximum of 500 letters are sent from each general practice clinic. If more than 500 patients are identified, the first 500 patients are randomly selected. The invitation letter directs prospective participants to an online survey to screen for eligibility criteria (Table 1). Questions in the screening survey are structured such that ineligible patients are redirected to a national social marketing website for healthy eating and physical activity promotion. Eligible patients reaching the end of the survey are able to nominate dates and times to attend an appointment with their GP at no cost to the patient. This 10 minute appointment is then booked for the patient and details of the appointment sent to the patient in a confirmatory short message service (SMS) text message.

**Figure 1:**
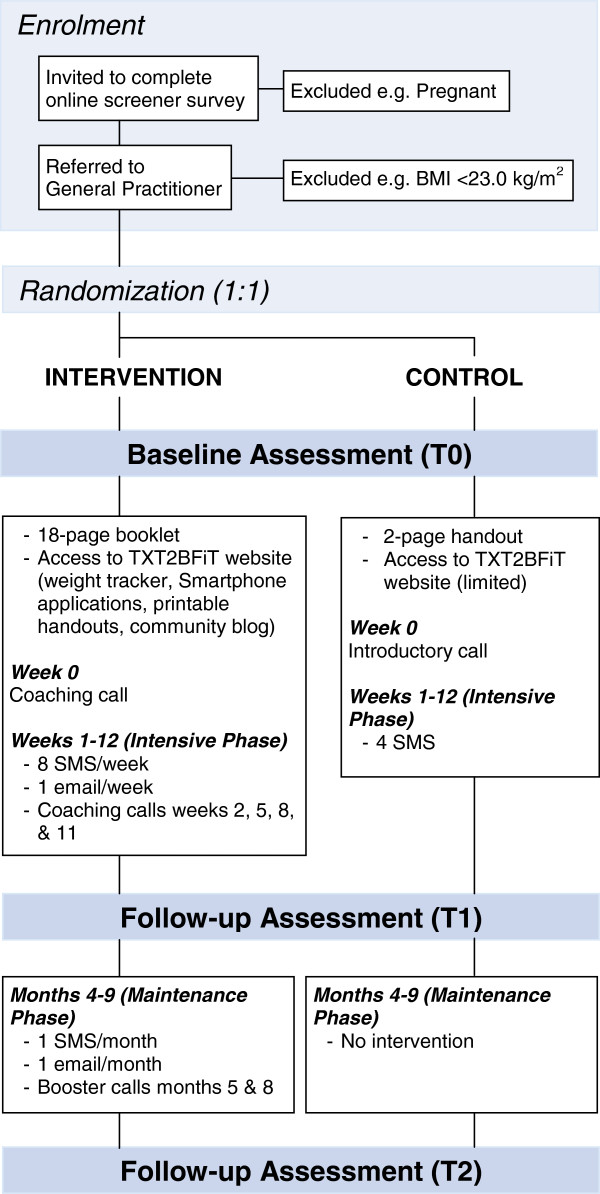
Diagrammatic summary of participant enrollment, randomization, assessment, allocated interventions, and associated time line.

At each appointment, the GP measures the patient’s weight and height and obtains the patient’s informed consent to participate in the trial by collecting their signed consent form. Signed consent forms are then faxed back to the researchers with the patient’s anthropometric data to confirm eligibility on the basis of their BMI and to enroll the patient in the trial. Enrolled patients are then randomized to the intervention or control arm by one of the researchers and allocated their treatment by another researcher by way of an introductory phone call (control participants) or a coaching call (intervention participants) provided in week 0. Figure 1 provides a diagrammatic summary of participant enrollment, randomization, assessment, and allocated interventions (intervention and control) with associated time line. While participants are aware of another arm to the trial, every attempt is made to ensure that the nature of this other arm is not revealed.

### Interventions

Control participants receive a mailed two-page handout summarizing the Australian national dietary and physical activity guidelines [24,25], an introductory call (week 0) to introduce the program (no coaching given), four SMS text messages (one every three weeks during weeks 1 to 12) that re-iterate information in the handout, and limited access to the TXT2BFiT website, that is, electronic versions of the two-page handout, consent form and study information statement, general information about the study and contact information. Intervention participants are mailed an eighteen-page booklet containing the two-page control handout, as well as sample meal plans, recommendations for daily servings from the core food groups with example serving sizes [25], and information about the four target behaviors addressed by the program: physical activity and sedentary behavior, intake of fruit and vegetables, intake of energy-dense take-away meals, and SSB intake, in conjunction with the TXT2BFiT mobile phone-based program.

#### TXT2BFiT program

This tailored program consists of SMS text messages, Emails, Smartphone mobile applications, a study website and personalized coaching calls (Figure 1). The program is grounded on processes-of-change theory [26], supported by self-monitoring, motivational interviewing and goal setting [27,28]. Through its various intervention components, the TXT2BFiT program consistently encourages participants to (1) consume five servings of vegetables and two servings of fruit per day, in line with the national dietary recommendations [25]; (2) limit energy-dense take-away meals to once per week or less [15]; (3) limit SSB to less than one liter per week [14]; (4) perform 60 minutes or more of moderate-intensity physical activity most days of the week (preferably daily), as evidence indicates that young adults are likely to require physical activity levels exceeding national guidelines to a level of approximately 45 to 60 minutes per day or 1.7 PAL (physical activity level; ratio of total to basal energy expenditure) to prevent weight gain [12,29].

### Study website

Each participant is provided with a unique login for the study website, to differentiate between the resources available to intervention and control subjects [30]. Throughout the nine month intervention, participants are able to access seven printable handouts to support physical activity, meal planning and selecting healthier foods and drinks when eating away from home; an interactive healthy weight tracker chart with green, yellow and red bars to indicate healthy, overweight and obesity, respectively, for self-monitoring changes in their BMI [31]; four Smartphone mobile applications developed to assist with forming self-monitoring skills for each target behavior [32]; and a community blog on which participants may post comments or questions to the researchers and communicate with other intervention participants.

### SMS text messages

Text messages are framed on the ten processes-of-change which define how one transitions from one stage-of-change to the next in the transtheoretical model for health behavior change [26]. Messages sent in week one are framed around the first process-of-change consciousness raising, which is aimed at ‘increasing information about self and problem…’ [26] in order to inform participants about how the target behaviors increase the risk for weight gain and recommendations for changing these behaviors to avoid weight gain. Subsequent text messages sent during the remaining 11 weeks of the intensive phase are tailored to the processes-of-change evident to be used in transitioning to action or maintenance from the participants’ baseline stage-of-change; that is, pre-contemplation (not thinking about change), intention (contemplation/preparation) or action (action/maintenance) [33-37]. For example, a greater proportion of text messages sent to participants in preparation for changing their fruit and vegetable intake are framed on self-reevaluation and helping relationships (that is, obtaining social support), as these processes are identified in the literature as assisting transition from intention to action for fruit and vegetable intake [34,36]. Fruit and vegetable messages coupled with replacing energy-dense foods, such as confectionery, crisps and energy-dense take-away foods with fruit or vegetables to promote a reduction in total energy intake for weight management [38]. Text messages are also tailored to gender, given the different motivators behind health behavior change experienced by men and women. For example, females may perceive a lack of exercise as conducive to weight gain, while males perceive this as conducive to weight loss (that is, muscle mass loss) [39]. Socio-cultural norms for young adults were also considered in developing the text messages. For example, young adults tend to use SSB as a mixer with alcohol and hence messages were developed to address moderating SSB consumption with alcohol [40]. Also considered were young adults’ perceived benefits and barriers to performing the desired behaviors, for example, lack of time [39], which was addressed by communicating convenient alternatives for healthy eating and physical activity. All text messages were sent using a two-way text messaging platform from a major commercial SMS service provider. Table 2 provides example text messages framed according to gender and processes-of-change consciousness raising and counter-conditioning (that is, ‘substituting alternatives for problem behaviors…’) [26].

**Table 2:** **Example mobile text messages framed according to gender and the processes-of-change *consciousness raising *and *counter-conditioning***

**Process-of-change**	**Gender**	**Fruit and vegetables**	**Physical activity**	**Sugar-sweetened beverages**	**Energy dense take-away meals**
**Consciousness raising**	***Males***	Fruit & Veg R high in fiber 2 keep U full & R low in fat. Stay Fit, without going hungry. Chicken breast & salad roll no butter (only 6 g Fat).	FACT: Physical activity improves mental alertness, reduces stress & depression & keeps U feeling energized. Plan 30mins of activity 4 tomorrow.	FACT: Sugary drinks give us a fast hit of energy. But just 1 hr after a sugary drink ur blood sugar levels drop affecting physical & mental performance.	FACT: Eating takeaway more than twice a week increases ur risk of gaining body fat & abdominal obesity (beer belly). TXT how many times a week U have takeaway.
	***Females***	FACT: Including Fruit & Veg makes it much easier 2 achieve & maintain a healthy weight. TXT how many Fruit & Veg U usually eat a day.	FACT: Women carrying excess weight R @ HIGH risk 4 further weight gain. Being active will STOP this weight gain. TXT the mins of activity U do in a week.	FACT: Sugary drinks give us a rush of energy from all the kilojoules. But just 1 hr after a sugary drink ur blood sugar levels drop causing U 2 feel tired & low.	FACT: Many takeaways contain over 1/2 ur daily kilojoules (kJ) needs. Check the kJ of ur meals: eTIYP mobile app @ http://txt2bfit.com/mobile-apps
**Counter-conditioning**	***Males***	Home late? U need an emergency kit: Pantry (tin corn, tomatoes, baked beans, tuna). Freezer (grainy bread, packet mixed Veg). Fridge (low fat cheese, eggs).	How many hrs do U spend online? Use ur mobile so U can walk while on the net. Avoid sitting by using stairs, walk don’t drive, cycle 2 work/study :-)	Do U use sugary drinks as a mixer 4 alcohol? U don’t have 2 miss out, just choose a diet (sugar free) drink as ur mixer.	THAI TIP: Avoid those fatty entrees. Try a hot chicken/beef/tofu salad with rice. Visit http://txt2bfit.com 4 more takeaway tips :-)
	***Females***	Always eating breakfast on the go? Choose a breakfast cereal with >5 g fibre/100 g & <20 g sugar/100 g, top with banana, strawberries & skim/low fat milk.	Eaten 2 much? Walk it off. 30mins tomorrow morning. Next time use a smaller plate, eat earlier & walk after. TXT ‘OK’ if U read this.	Craving guilt free enjoyment? Quench ur thirst with water. Add a twist of lemon 4 a hit of VitaminC. Leave the lemon in your bottle & fill up 4 FREE.	Planning takeout & TV tonight? Walk 2 the shops instead: frozen stir-fry vegies, beef OR tofu, soy sauce, garlic & rice.

### Coaching calls

The aims of the coaching calls are to set personalized goals with participants, educate them on healthy eating and physical activity for weight management, address personal barriers to behavior change, maintain motivation and notify participants of, and encourage them to use, the additional program materials available. At each coaching call, participants are asked about their current body weight, diet and physical activity in order to set goals to be achieved by their next coaching call (approximately two or three weeks), as setting proximal dietary and weight-related goals may be more effective than distal (long-term) goals [27]. During each call, one goal is set with participants for their body weight and between one and three goals for their diet or physical activity (around the target behaviors or other weight-related aspect of their diet or physical activity). Coaching calls are delivered by a dietitian with regular quality checks by an external researcher listening to calls in order to ensure adherence to pre-specified procedures.

### Emails

Emails sent to intervention participants act as reminders to use the program materials available, and provide an additional source of education on healthy eating, physical activity, goal setting and self-monitoring behaviors.

### Outcome measures

Measurements are taken at baseline (T0), three months (T1, week 13) and nine months (T2, week 37) (Figure 1). At each time point, participants complete an online survey to assess weight; height; stage-of-change; well-being; intake of fruit, vegetables, energy dense take-away meals, SSB, and water; knowledge of fruit and vegetable recommendations; physical activity; sitting time; and self-efficacy.

#### Demographics

At baseline, all participants are asked their age, gender, ethnicity (language spoken at home [41]), postcode (for categorizing socio-economic status [42]), education level [41], living arrangement, income bracket [41], and number of hours spent in work or study per week.

#### Primary outcomes

Body weight is measured to the nearest 0.1 kg at baseline by the GP and again after three months by study personnel blinded to participant allocation using WHO protocols [31]. Self-reported weight is collected at baseline, three months and nine months via an online survey. BMI is calculated as weight (kg) / [height (metres)^2^ at baseline, three months and nine months.

#### Secondary outcomes

Frequency of consuming specific foods and drinks (fruit, vegetables, energy-dense take-away meals, SSB, and water) is assessed using short dietary questions [43,44]. Knowledge of fruit and vegetable recommendations is assessed using the question ‘What do you think is the recommended number of serves of (fruit/vegetables) that should be eaten in a day?’ adapted from Pollard *et al*. [45]. Multiple choice response options are ‘one serve a day’, ‘two or three serves a day’, ‘four or five serves a day’ and ‘six or more serves a day’ for fruit and ‘one or two serves a day’, ‘three or four serves a day’ and ‘five or more serves a day’ for vegetables. Intake of specific nutrients (energy, protein, total fat, saturated fat, carbohydrate, sugars, dietary fiber, and alcohol) is assessed using an online food frequency questionnaire, the Dietary Questionnaire for Epidemiological Studies version 2 (DQES v2), administered at baseline and three months [46]. Stage-of-change for the target dietary behaviors (fruit, vegetables, energy-dense take-away meals and SSB) is assessed by firstly measuring the target behavior, for example, serves of vegetables eaten per day. Participants reporting the desired behavior (that is, vegetable serves five or more per day; fruit serves two or three per day; energy-dense take-away meals ≤ once per week; SSB < 1 L per week) are directed to the question: ‘Have you been eating/drinking this amount of (behavior) for more than six months?’ to classify them as being in either action (performing the desired behavior for ≤ six months) or maintenance (performing the desired behavior for > six months). Participants not performing the desired behavior are asked: ‘Are you thinking about eating/drinking more/less (behavior) sometime in the next six months?’ to classify those in contemplation/preparation (intent on changing in ≤ six months) or pre-contemplation (not intent on changing in ≤ six months). Participants intent on changing in ≤ six months are further asked: ‘Are you planning to eat/drink more/less (behavior) sometime in the next month?’ to discriminate between those in preparation (intent on changing in ≤ one month) or contemplation (not intent on changing in ≤ one month).

Physical activity and time spent sitting in the previous seven days are self-reported using the International Physical Activity Questionnaire [47], and the domain specific sitting time questionnaire [48], respectively. Stage-of-change for physical activity behavior is assessed using the question: ‘Thinking about the last two weeks, on average, did you do physical activity for at least 60 minutes each day?*’* adapted from Marcus *et al*. [49], with response options ‘Yes, and I have been doing this for more than six months’ (maintenance), ‘Yes, but I have been doing this for less than six months’ (action), ‘No, but I intend to in the next 30 days’ (preparation), ‘No, but I intend to in the next six months’ (contemplation), ‘No, and I do not intend to in the next six months’ (pre-contemplation).

Self-efficacy in performing each of the target behaviors is assessed with the question ‘How confident are you that you can…(perform the target behavior, for example, eat at least five serves of vegetables each day (one serve = one cup salad OR half a cup cooked vegetables))?’ with response options on a four-point Likert scale from ‘Not at all confident’ to ‘Very confident’ adapted from the Project-Eat II survey for young adults [50]. Well-being is assessed using the WHO-5 well-being index which assesses five items of subjective well-being in the past two weeks on a six-point Likert scale ranging from ‘All of the time’ to ‘At no time’ [51].

### Engagement, reach and implementation

Measures of engagement with the program include number of text message replies, completed coaching calls, logins to Smartphone applications, and posts on the community blog. These data are collected continuously throughout the nine month trial. Intervention participants are asked to reply ‘OK’ to one text message every three weeks during the intensive phase (weeks 1 to 12) and to each text message sent during the maintenance phase (weeks 14 to 36), while control participants are asked to reply ‘OK’ to all four text messages sent during the intensive phase. Online surveys administered at T1 and T2 follow-up assessments ask participants (intervention and control arms) about their use of, and any problems experienced with, program materials. Socio-demographic data of participants will be used to assess program reach within Medicare Locals. Costs of developing and delivering the program are recorded. The method of recruitment via general practice clinics will also be evaluated for potential as a setting for referring young adults into the program through structured interviews with practice staff involved in the trial.

### Sample size

The average weight loss of young adults participating in an intervention aimed at weight gain prevention is 0.87 kg (95% confidence interval (CI) -1.56, -0.18), while controls gain on average 0.86 kg (95% CI 0.14, 1.57) [19]. To detect a mean difference of 2.0 kg with *P =* 0.05 and 80% power, assuming a standard deviation of 10 kg and a correlation between baseline and final weight of 0.8, 284 subjects are required (142 per arm). Allowing for a 20% drop out, 354 subjects are needed (177 per arm). This number will be sufficient to detect a change in fruit and vegetables of 0.5 serves per day, a 20% change in the proportion having energy-dense take-away meals once a week or less, and an increase in physical activity of 60 minutes per week.

### Randomization

Eligible patients are randomized in a 1:1 ratio into intervention and control arms. Randomization is based on a stratified randomized block design, where the strata are the Medicare Local, general practice clinic, and participant gender. The random sequence will be generated by an independent researcher and concealed from those responsible for enrolling and assigning interventions to patients.

### Statistical analysis

The primary outcome, body weight at three months, will be compared between the two groups using analysis of covariance adjusting for baseline weight and the stratification variables Medicare Local, general practice clinic and participant gender. The analysis will be by ‘intention-to-treat’ principle using multiple imputation to account for missing data. Secondary outcomes that are continuous will also be analyzed using analysis of covariance, while Chi-squared tests will be used to analyze dichotomous outcomes. Mixed models will be used to describe changes in outcomes over time. Personnel analyzing participant outcomes will be blinded to participant allocation. Further sensitivity analyses will be conducted using measures of participant engagement with program materials.

## Discussion

Young adults are an often forgotten population group for obesity prevention programs. It is uncommon for young adults to be counseled on healthy eating or physical activity in the interest of preventing weight gain in primary care [52]. This may be due to limited awareness of weight gain as a health issue for young adults among practitioners and patients, or time constraints on consultations that limit practitioner-patient discussions to the reason for encounter (which for young adults is likely to be a general health check, respiratory complaint, feedback on test results, or psychological issue) [53]. Currently, there exists a gap in primary health care and broader community obesity prevention strategies to address unhealthy weight gain in young adults. The development and testing of services or programs that will be acceptable and appropriate to this demographic is urgently required to address this gap in preventative health care.

Existing mobile phone-based programs for weight management developed for other population groups have largely consisted of SMS text messaging and/or Smartphone mobile applications. Programs based on text messaging have been demonstrated to be effective for reducing body weight, waist circumference and increasing physical activity in adults, and for promoting adherence to monitoring intake of SSB, physical activity and screen time in children [54-56]. Efficacy of mobile phone-based programs may be enhanced by tailoring text messages to the individual, providing education on the target behavior, and providing additional forms of intervention [55,57]. Hence, the TXT2BFiT program tailors text messages to the individual’s baseline stage of change, provides education on the target behaviors (through printable handouts, text messages and personalized coaching phone calls), and provides additional intervention through Email, online resources and the coaching phone calls.

A potential adverse effect of mobile phone-based interventions with young adults may be increased stress as mobile phone use has been identified as a source of stress for some young adults. These stresses stem from demands on making one’s self available at all times for work and social communication, disturbing rest periods and complicating the separation of work or study from private life [58]. This was addressed in the protocol by only sending SMS messages to participants between the hours of 11:00 and 18:00, not requiring participants to reply instantaneously to text messages, and offering flexibility in dates and times to receive coaching phone calls.

The current protocol is consistent with the best available evidence regarding intervention strategies that are likely to be effective for preventing weight gain among this population, as the duration is ≥ four months, the program addresses physical activity, dietary energy density and behavioral skills for weight management (such as self-monitoring and goal setting), and program materials are tailored to the individual through personalized coaching calls and SMS messages framed around the individual’s stage of change [19]. While the dynamicity of electronic communication technologies presents a challenge to the sustainability of all health interventions delivered via these technologies, the basic behavior change principles and strategies used in the TXT2BFiT program may be translated to newer delivery platforms if necessary.

An important aspect to the design of public health interventions is the potential reach of the program to the intended audience. The benefit of the TXT2BFiT program is that it may be delivered to anyone with an active internet connection and mobile phone, and reach of the program to trial participants will be evaluated using demographic data of the study participants. Recruitment is limited to urban areas of Sydney, New South Wales, but this will provide a sample of varied ethnic and socio-economic backgrounds, considering most research to date in this population has been restricted to highly educated samples [19]. Prospective participants are volunteers who are likely to be more highly motivated, although this is more a limitation for generalizability to less motivated young adults than selection bias, given the RCT design of the trial.

The mobile phone-based program described addresses an important gap in obesity prevention efforts to date, given the risk for future weight gain among young adults. The method of intervention delivery is via platforms that are highly accessible and appropriate for this population group. The program has capacity to be delivered by minimal staff with flexibility in setting for program delivery. If effective, further translational research will be required to assess the practical aspects of how this program might operate if made available to young adults in the broader community.

### Trial status

The TXT2BFiT trial was designed in 2011-12 and is currently in its recruitment phase which is anticipated to continue until late 2013. Funding was received in January 2012 and ethical approval obtained in September 2012.

## Abbreviations

ACTRN: Australian New Zealand clinical trials registry numberBMI body mass index; CI: Confidence interval; DQES v2: Dietary questionnaire for Epidemiological Studies version 2; GP: General practitioner; PAL: Physical activity level; RCT: Randomized controlled trial; SD: Standard deviation; SMS: Short message service; SSB: Sugar sweetened beverages; WHO: World Health Organization.

## Competing interests

The authors declare they have no competing interests.

## Authors’ contributions

All authors were involved in the conception of the trial design and reviewing the final manuscript; ED-W and MH informed the recruitment strategy; KMcG informed the sample size calculations and analysis methods; ethical approval was sought by KB, LH and MAF. All authors read and approved the final manuscript.
